# Quantitative, In Situ Visualization of Metal‐Ion Dissolution and Transport Using ^1^H Magnetic Resonance Imaging

**DOI:** 10.1002/anie.201604310

**Published:** 2016-06-22

**Authors:** Joshua M. Bray, Alison J. Davenport, Karl S. Ryder, Melanie M. Britton

**Affiliations:** ^1^ School of ChemistryUniversity of BirminghamBirminghamB15 2TTUK; ^2^ School of Metallurgy and MaterialsUniversity of BirminghamBirminghamB15 2TTUK; ^3^ Materials CentreDepartment of ChemistryUniversity of LeicesterLeicesterLE1 7RHUK

**Keywords:** batteries, copper, corrosion, electrochemistry, magnetic resonance imaging

## Abstract

Quantitative mapping of metal ions freely diffusing in solution is important across a diverse range of disciplines and is particularly significant for dissolution processes in batteries, metal corrosion, and electroplating/polishing of manufactured components. However, most current techniques are invasive, requiring sample extraction, insertion of an electrode, application of an electric potential or the inclusion of a molecular sensor. Thus, there is a need for techniques to visualize the distribution of metal ions non‐invasively, in situ, quantitatively, in three dimensions (3D) and in real time. Here we have used ^1^H magnetic resonance imaging (MRI) to make quantitative 3D maps showing evolution of the distribution of Cu^2+^ ions, not directly visible by MRI, during the electrodissolution of copper, with high sensitivity and spatial resolution. The images are sensitive to the speciation of copper, the depletion of dissolved O_2_ in the electrolyte and show the dissolution of Cu^2+^ ions is not uniform across the anode.

In many electrochemical experiments, it is often assumed that the total measured current is distributed uniformly across the entire electrode surface. Any evidence for an inhomogeneous distribution usually comes from post‐mortem examination of electrode surfaces, which typically requires the system to be dismantled. This is a destructive process and can be, in the case of some batteries, potentially dangerous. Hence, there has long been considerable interest in the development of non‐invasive, in situ measurements of local current distribution. One of the biggest challenges, in this respect, is the detection of the distribution of metal ions in solution, which is critical for the development of improved battery, anti‐corrosion, and electroplating technologies.

The detection of metal ions in solution can be performed either spectroscopically or electrochemically. In situ electrochemical detection typically uses ion‐selective electrodes or scanning electrochemical microscopy^1^ (SECM), which is often combined with anodic stripping voltammetry^2^ (ASV) to detect metal‐ion concentrations at the interface between the electrolyte and metal with a spatial resolution on the order of tens of microns at concentrations in the parts per billion (ppb) to parts per trillion (ppt). However, as the sample must be scanned relative to an electrode tip, the technique is invasive, resulting in the disturbance of mass transfer profiles, and images can be relatively slow to collect, cover a relatively small area (on the order 10^2^×10^2^ μm^2^) and are generally limited to a two‐dimensional (2D) region within the diffusion layer. In situ spectroscopic monitoring of electrochemical reactions^3^ has employed a variety of techniques, including UV/Vis, infrared (IR), and Raman spectroscopies. The spectroscopic detection of metal ions is most commonly achieved through the use of molecular sensors,^4^ which are typically probed using fluorescence spectroscopy or microscopy, enabling detection of the presence, concentration and environment of metal ions through a modulation of the life time, anisotropy or intensity of the fluorescence signal of the molecular probe. Optical methods cannot be used on optically opaque samples and concerns arise that molecular sensors may affect the chemistry of the system under study. Nuclear magnetic resonance (NMR) avoids both of these difficulties as it is able to detect the presence and concentration of metal ions in solution, either directly for NMR‐active nuclei such as ^7^Li or ^23^Na or, indirectly, through the measurement of ^1^H NMR relaxation times of solvent molecules,^5^ which can be sensitive to the presence and speciation of metal ions. In magnetic resonance imaging (MRI), this information becomes spatially resolved through the use of magnetic field gradients.5b However, there are very few MRI studies mapping the distribution of metal ions near bulk metals.^6^ This is largely due to difficulties with performing MRI experiments on samples containing bulk metals, as a result of the presence of magnetic susceptibility artefacts and generation of eddy currents in the bulk metal.^7^ While, recent studies have shown that it is possible to overcome these problems by careful control of electrode orientation and cell geometry,^7^, ^8^ another limitation, is that there are very few nuclei that can be readily imaged by MRI. In fact, the vast majority of nuclei are difficult, or impossible, to directly image with MRI, due to low sensitivity and abundance, as well as short *T*_2_ relaxation times. Hence, indirect detection, by ^1^H MRI, offers a unique opportunity for insight into many more systems of interest than are currently accessible. Also, as ^1^H has high abundance and sensitivity, quantitative, 3D mapping can be performed in real time.

Herein, we demonstrate the first example of ^1^H MRI to produce quantitative, 3D concentration maps of non‐MRI‐observable ions, in this case copper ions, going into solution from a bulk metal, in real time. We use the electrodissolution of copper as a test system because bulk copper has a low magnetic susceptibility and Cu^2+^ ions are paramagnetic, hence detectable at low concentrations. Furthermore, the corrosion of copper is of substantial industrial and societal interest owing to the economic and safety implications associated with the widespread use of copper and its alloys in domestic and industrial heating/cooling systems, electronics, fuel distribution piping, battery current collectors, medical components and nuclear waste storage. Experimental studies of copper corrosion have largely relied on bulk electrochemical measurements,^9^ such as cyclic voltammetry, analysis of the metal surface and bulk identification of corrosion products,^10^ typically using energy dispersive X‐rays (EDX) and Raman spectroscopy. Recently, SECM with underpotential anodic stripping voltammetry (UP‐ASV) has been used to image Cu^2+^ ion release during the corrosion of copper deposits on metal surfaces.2e This study visualized electrochemically active sites in 2D, over a scan area of 215×160 μm^2^ at 15 μm resolution. While this method is able to detect Cu^2+^ with a detection limit of 0.15 nm, it is not able to produce quantitative concentration maps of Cu^2+^ and, as with other SECM techniques, is invasive. Also, being limited to a relatively small 2D area, it requires pairing with an AC‐SECM scan to identify a region of interest—a combined process taking over an hour to perform. Anodic dissolution of copper has also been investigated using digital holography.^11^ While this technique is rapid and benefits from being non‐invasive, it is unable to quantify copper ion concentrations or map the surface in 2D. Thus, as MRI is able to non‐invasively map the distribution of metal ions^7^ in 3D, in situ and in real time, it offers a unique and novel opportunity to investigate the underlying mechanisms of corrosion as well as underpin investigations on the action of corrosion inhibitors.

MRI quantification of dissolved Cu^2+^ concentrations is possible through measurement of ^1^H NMR relaxation times of solvent (water) molecules, which are sensitive to the presence and concentration of paramagnetic Cu^2+^ ions. A plot showing the linear relationship between relaxation rate (1/*T*_1_) and Cu^2+^ concentration is shown in Figure 1. The relaxivity of Cu^2+^ in aqueous solution was determined from the slope of this plot, giving 0.712±0.001 mm ^−1^ s^−1^ at 7 T. The low concentration region of the plot in Figure 1 b indicates a nominal detection limit for Cu^2+^ ions of about 20 μm. Figure 2 a shows a schematic diagram of the cell used in the MRI magnet; a description of the experimental set‐up is provided in the supplementary information. By aligning the cell with respect to the static magnetic (*B*_0_) and radiofrequency (*B*_1_) fields, as shown in Figure 2 a, it is possible to collect distortion‐free images of the electrolyte between two copper electrodes. It was discovered that in addition to the alignment constraints for the cell with respect to *B*_0_ and *B*_1_ fields, it was also necessary to restrict the height of the fluid between the electrodes to maintain *B*_1_ field homogeneity (see the Supporting Information). Figure 2 b shows 2D horizontal (*xy*) and vertical (*xz*) images (with a slice thickness encompassing the full width/height of the cell).

**Figure 1:**
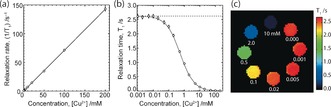
a) A plot of relaxation rate, 1/*T*_1_, (circles) for water in 0.5 m Na_2_SO_4_, as a function of CuSO_4_ concentration, with a linear fit to the data. b) The relaxation time data for the plot in part (a) with the concentration plotted on a logarithmic scale. The dashed line shows the relaxation time of the electrolyte in the absence of Cu^2+^ ions. c) A ^1^H MRI *T*_1_ relaxation map for a series of 5 mm NMR tubes containing 0.5 m Na_2_SO_4_ with varying CuSO_4_ concentrations. In this image, the 10 mm concentration sample, which has a *T*_1_ of 0.14 s, appears almost black.

**Figure 2:**
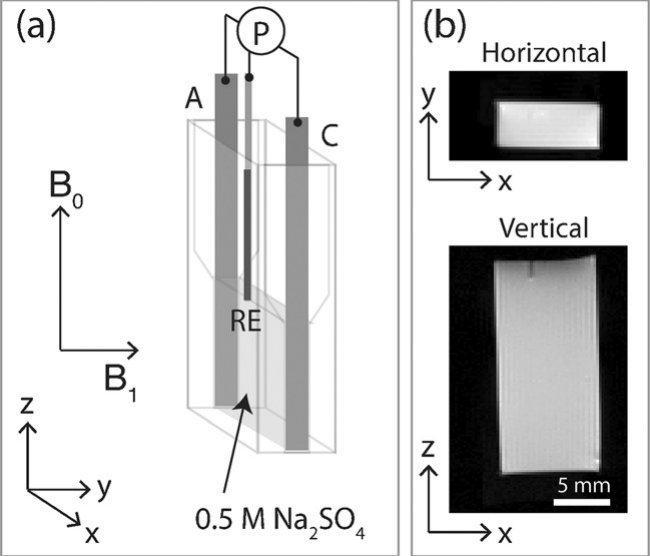
a) Schematic diagram of the MRI corrosion cell, showing the anode (A), cathode (C), and Ag/AgCl reference electrode (RE), along with their connections to potentiostat (P). b) Vertical and horizontal ^1^H MR images of a 0.5 m Na_2_SO_4_ electrolyte in the electrochemical cell. In the vertical image, the Ag/AgCl reference electrode tip is just visible at the top of the cell as a small vertical black line. The slice thickness for the horizontal image is the full depth of the cell (along *z*) and the slice thickness for the vertical image is the full thickness of the cell (along *y*).

A series of *T*_1_ relaxation maps of the electrolyte in the cell were collected while the cell was inside the MRI magnet (Figure 3 a), during controlled galvanostatic electrodissolution of the copper anode. The leftmost image was collected before the first current pulse and shows that the electrolyte has a uniform *T*_1_ relaxation time (*T*_1_=2.62 s) equal to that expected for the 0.5 m Na_2_SO_4_ electrolyte, indicating that if there is any Cu^2+^ in solution it is below the level of detection (<20 μm). Each subsequent map was acquired following application of a fixed current (as indicated in Figure 3), for a controlled time, after which images were acquired with the cell at open circuit. Using the relaxivity of dissolved Cu^2+^ ions, the *T*_1_ maps were converted into corresponding Cu^2+^ concentration maps (Figure 3 b). These maps show Cu^2+^ ions going into solution at the anode and propagating towards the cathode. The vertical maps show that dissolution occurs predominantly at the bottom of the strip. It was investigated whether the build‐up of Cu^2+^ ions at the bottom of the anode was caused by density‐driven convection. Examination of the distribution of Cu^2+^ ions, following an application of a current pulse, indicated no significant change in the distribution of Cu^2+^ over time, other than that expected from diffusion. Also, NMR measurements of diffusion did not show any enhancement in the mobility of solvent molecules. Hence, if gravitational convection is present, it is a weak effect, and the inhomogeneous distribution of Cu^2+^ ions is more likely due to inhomogeneous electrodissolution.

**Figure 3:**
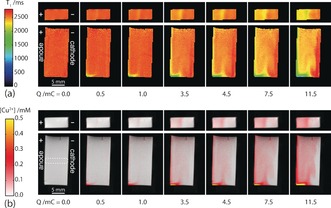
a) *T*_1_ relaxation time maps from the horizontal (top) and vertical (bottom) images, with the corresponding total net charge passed (*Q*) indicated below. b) Concentration maps of Cu^2+^ from the *T*_1_ maps shown in (b). The dashed box indicates a region of the cell used to produce the concentration profiles presented in Figure 4.

To test the quantitation of these maps, the total number of moles of dissolved Cu^2+^ measured in the cell was compared (Figure 4 a) with the charge passed (*Q*) and the corresponding moles of copper ions expected, $n_{{\mathrm{Cu}}^{2+}}$ , from Faraday's law ($n_{{\mathrm{Cu}}^{2+}}=Q/\;2F$ ; where F is Faraday's constant). Good agreement is observed between the total numbers of moles Cu^2+^ measured from the horizontal and vertical images. Up to a cumulative charge of 5 mC, the relationship between moles of Cu^2+^ and electrons follows a ratio of 2 moles of electrons to 1 mole of Cu^2+^, indicating the MRI results are highly quantitative.

**Figure 4:**
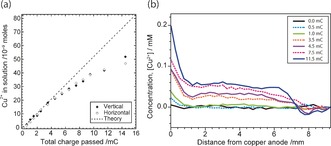
a) Plot of the total number of moles of Cu^2+^ ions measured experimentally from the vertical and horizontal MRI images as a function of the total charge delivered by the potentiostat. The dashed line represents the expected 1:2 ratio of moles of Cu^2+^ ions to electrons. b) Plot of averaged concentration profiles from the region indicated in the image shown in Figure 3 b. The anode is located at position 0.0 mm and the cathode at 9.2 mm.

However, after a total charge of 5 mC has passed, the moles of Cu^2+^ appear to be progressively underestimated as more current is applied. This underestimation of the moles of Cu^2+^ appears to be linked to the sharp drop in [Cu^2+^] detected near the cathode, which can be best observed in the series of concentration profiles presented in Figure 4 b. These profiles were collected from the region in the middle of the cell indicated in Figure 3 b, away from the area of increased dissolution at the bottom of the cell. These profiles show there is an increase in dissolved Cu^2+^ as each current pulse is applied, and the concentration is highest by the anode. The profiles at a cumulative charge of 3.5 mC, and above, show a sharp drop in the concentration of Cu^2+^ near the cathode, which in the case of the profile at 11.5 mC even becomes negative. This drop in concentration of Cu^2+^ is expected to be associated with the reduction of dissolved O_2_ at the cathode producing hydroxide ions,^12^ which leads to an increase in pH (Figure SI4 in the Supporting Information), and the subsequent formation of Cu(OH)_2(s)_ or Cu_2_O_(s)_ near the cathode.^13^ As these Cu species are solid, they no longer affect the relaxation of surrounding water molecules and hence become MRI‐invisible. The apparently negative concentration of Cu^2+^ at 11.5 mC is a consequence of the change in relaxation time for the solvent associated with the removal of oxygen by the cathodic reaction. As the oxygen molecule is paramagnetic, its removal from the solution results in an increase in the relaxation time, and hence a decrease in the relaxation rate, of the solvent (see the Supporting Information).

Finally, our ability to visualize the distribution and concentration of Cu^2+^ ions in 3D is demonstrated in the images shown in Figure 5. These images were acquired from a second sample after 2 and 16 mC of charge had been passed and, with an acquisition time of 25 s, show the evolution of Cu^2+^ in solution in real time. In this second sample, a scratch was applied to the anode, immediately prior to filling the cell with electrolyte, in order to give this local region a thinner oxide film. In the images in Figure 5, a region of increased dissolution at the bottom of the electrode can be seen, as was observed for the previous sample (Figure 3). However, there is also a region of increased dissolution in the centre of the anode, at the position of the scratch. This central active region, which has the same shape as the scratch, can be best observed in the 2D images of the electrolyte layer (500 μm thick) immediately adjacent to the anode surface shown in Figure 5 b,d. Thus, these images highlight the ability of the technique to image heterogeneous dissolution across the copper surface.

**Figure 5:**
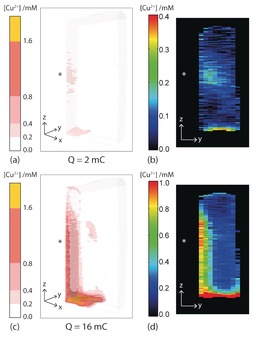
3D (a,c) and 2D (b,d) concentration maps of Cu^2+^ ions dissolved in 0.5 m Na_2_SO_4_ electrolyte following the electodissolution of the Cu anode. The images in (a,b) was acquired after delivery of 2 mC of charge and the images in (c,d) after 16 mC of charge. The pixel size is 500 μm(*x*)×500 μm(*y*)×188 μm(*z*), and the asterisk indicates the region on the anode that was scratched immediately prior to filling the cell with electrolyte. The 2D images in (b) and (d) are of the electrolyte layer (500 μm thick) immediately adjacent to the anode surface, and were extracted from the 3D maps shown in (a) and (c), respectively.

In conclusion, we have demonstrated that ^1^H MRI can be used to non‐invasively visualize—quantitatively, in 3D and in real time—the electrochemical generation and transport of metal ions that are not accessible directly using MRI, through analysis of the ^1^H MR signal from the solvent in the electrolyte. The MR images identified the presence of regions of enhanced localized dissolution across the copper foil. As ^1^H MR images can be acquired on the order of seconds, it is possible to follow metal‐ion dissolution processes in real time. This methodology is able to achieve a spatial resolution on the order of 10^−5^ m, which is comparable with current SECM methods, but is able to cover a significantly larger area (ca. cm^2^) and is non‐invasive. By comparing the measured amount of Cu^2+^ in the cell with the charge passed, we have shown that the technique is quantitative and able to accurately detect Cu^2+^ ions in solution, with a sensitivity of ±20 μm. The technique is also sensitive to the change in speciation of Cu in the region of the cathode where there is an increase in pH as a consequence of the reduction of dissolved O_2_ in the electrolyte. Thus, this method provides important insights into both mass transfer and speciation of metal ions. While the electrodissolution of copper was used as a test system, preliminary experiments indicate this technique can also be applied to the study of other electrodissolution processes, such as those found in Al–air batteries, Zn electroplating or the corrosion of austenitic (non‐magnetic) stainless steel.

## Supporting information

As a service to our authors and readers, this journal provides supporting information supplied by the authors. Such materials are peer reviewed and may be re‐organized for online delivery, but are not copy‐edited or typeset. Technical support issues arising from supporting information (other than missing files) should be addressed to the authors.

Supplementary
