# Supplementary material for: Quantitative, In Situ Visualization of Metal‐Ion Dissolution and Transport Using 1H Magnetic Resonance Imaging
Source: Angew Chem Int Ed Engl. 2016 Jun 22;55(32):9394–7. doi: 10.1002/anie.201604310 (PMC5094501; doi:10.1002/anie.201604310)
Supplement: Supplementary file 1 — Supplementary [file ANIE-55-9394-s001.pdf]

Angewandte Chemie
Eine Zeitschrift der Gesellschaft Deutscher Chemiker

Supporting Information

Quantitative, In Situ Visualization of Metal-Ion Dissolution and Transport Using ^{1}H Magnetic Resonance Imaging

*Joshua M. Bray, Alison J. Davenport, Karl S. Ryder, and Melanie M. Britton**

anie_201604310_sm_miscellaneous_information.pdf

SUPPLEMENTARY INFORMATION

Contents

1. Effect of metal ions on solvent relaxation times
2. Methods
3. Artefacts in MRI imaging near metal electrodes
4. Cu^{2+} Concentration calibration
5. Optical images showing change in pH using Universal Indicator
6. Electrochemistry
7. Concentration calibration for 3D mapping

1. Effect of metal ions on solvent relaxation times

Magnetic resonance relaxation times of solvent molecules are affected by the presence and concentration of metal ions. This effect is most apparent for paramagnetic ions, but is also observed in diamagnetic ions. Investigation of the relationship between relaxation time and metal ion concentration has mostly focussed on aqueous solutions[1,2], though there are some studies of non-aqueous solutions also[2]. In the case of diamagnetic ions, the effect is mostly due to changes in the mobility of the solvent, through viscosity changes and co-ordination of the water molecules with the metal ion. Whether there is an increase or decrease in the mobility of the solvent molecules, caused by the metal ion, will dictate whether the relaxation time decreases or increases, respectively, with increasing metal ion concentration. Typically, molar quantities of the metal ion are required before an effect on the relaxation time between apparent.

The T1 and T2 relaxation times of water molecules are greatly affected by the presence of paramagnetic ions, such as Cu^{2+}. Relaxation becomes very efficient in the presence of paramagnetic species, due to the magnetic moment of unpaired electron spins, which is 1000 times greater than nuclear magnetic moments. Hence, only micro- to millimolar concentrations are, typically, required before a reduction in the relaxation time is observed[3].

2. Methods

Electrochemical cells. The corrosion cells were constructed from a polystyrene semi-micro cuvette (Sarstedt, Germany) with two copper (99.98%, Aldrich) electrodes, of dimensions 0.25 × mm 51.0 mm × 3.7 mm, and a 0.50 mm diameter chloridized silver wire (Ag/AgCl) reference electrode (RE). The copper electrodes were cleaned in 10% nitric acid for 1 minute, then rinsed in nanopure water, then methanol, before left to dry for over 16 hours. Electrodes were fixed to the cuvette walls by slightly dissolving the plastic cuvette with a small drop of chloroform, leaving only the front face exposed with no air bubbles in contact with the walls. The RE was held in position using a silicone plug inside the cell, and a measurement of the RE potential pre/post experiment in 3 M NaCl relative to a conventional Ag/AgCl reference electrode (BASi, USA) showed that it drifted by –1.4 mV. The electrolyte was 0.5 M Na_2SO_4 (> 99.0%, Sigma), prepared in ultrapure water, at pH 5.5. A 750 μL volume was injected just prior to insertion of the cell into the MRI. For the 3D mapping experiment shown in Figure 5, the electrodes in this cell were exposed to the atmosphere for 1 week to allow formation of an oxide layer, and just before introduction of the electrolyte, a small scratch was made near the centre of the anode using a needle.

MRI. ^{1}H MRI was performed on a Bruker DMX300 spectrometer, operating at a ^{1}H resonance frequency of 300.13 MHz and equipped with a 25 mm micro2.5 radiofrequency (RF) resonator, at ambient temperature of 19 ± 0.2 °C. The RF coil and corrosion cell were independently rotated in-situ to align the strip electrodes so that they were parallel with the B_0 and B_1 fields.

A series of vertical (*xz*) and horizontal (*xy*) 2D images (projections) were acquired using a RARE imaging sequence[4] to produce spin density and T_1 maps of protons in the electrolyte solution. Vertical images were acquired with a 25 mm slice thickness,

a field of view (FOV) of 24 mm × 16 mm, a pixel size of 188 μm × 250 μm and RARE factor of 64. Horizontal images were acquired with a 50 mm slice thickness, a FOV of 16 mm × 8 mm, a pixel size of 250 μm × 250 μm and RARE factor of 32. Slice thicknesses were sufficiently large to ensure the entire sample was contained within each image, producing 2D images (projections) of the electrolyte. T_1 images were produced from eight 2D images with an inversion recovery delay ranging from 5 ms to 14 s, all with an echo time of 3.2 ms and a recovery time of 14.1 s, and taking 8 minutes in total to acquire. The 3D concentration maps were acquired from a single 3D image, acquired with a FOV of 24 mm × 16 mm × 8 mm, with a pixel size of 188 μm × 500 μm × 500 μm, a RARE factor of 16, a recovery time of 200 ms and resulting in an acquisition time of 25 s. In the 3D maps, the concentration was determined from image intensity, which is a function of the local T_1 relaxation time and was calibrated by imaging known copper concentrations using identical imaging parameters (see supplementary information for more details).

Potentiostat. A COMPACTSTAT.e potentiostat (Ivium), in 3-electrode mode, controlled the electrochemical cell inside the MRI, and was connected to the cell electrodes using low magnetic susceptibility RG316 coaxial wires (Radiall). Electromagnetic noise was minimised using in-line low pass filters (DC to 48 MHz pass band; Mini Circuits, Brooklyn, NY) and an 8 mm thick copper bulkhead plate was used to cover the bore entrance for shielding. The following protocols were used: 1) the open circuit potential (OCP) was measured during MRI scanning (1120 s acquisition); 2) a galvanostatic current of 50 μA or 100 μA was applied for a fixed time (ranging between 10 and 60 s) to produce controlled dissolution of the anode. The OCP was measured and remained in the range E = –30 mV to +150 mV. With the cell connected to the potentiostat and loaded into the MRI, the OCP was monitored while MRI setup was conducted and there was no indication of any perturbation of the potential during MRI measurements. Once the voltage had stabilised, a data set was acquired before any current was passed through the cell. At each experimental point, the charge pulse was delivered as described above, followed immediately by the same concurrent OCP/MRI acquisition.

Optical photography. An electrochemical cell was fashioned as above using a semi-micro cuvette with one plastic sidewall removed to reveal the internal, clear plastic window for unobstructed view of the fluid inside. The opposite sidewall was

roughened with 1200 grit SiC paper to a smooth/translucent finish to permit diffuse but uniform backlighting. A 0.50 M Na_2SO_4 electrolyte was mixed using 6 %-vol Universal Indicator (Fluka). The cell was clamped in place, and photography was done using a digital camera (Canon PowerShot SX600 HS) on a tripod, with a darkened room and a backlit, white paper background used for uniform, reproducible lighting. Controlled anodic dissolution was done using a series of galvanostatic pulses (0.1 mA, variable duration), each followed by a 120 s OCP measurement.

3. Artefacts in MRI imaging near metal electrodes

Accurate determination of ion concentrations from relaxation maps depends on acquiring accurate, distortion-free images that are used to produce those maps. Image artefacts may arise from distortion of the primary magnetic field (B_0) due to the magnetic susceptibility of the metals or by interaction between the radiofrequency (RF) field and the electrodes. The RF field may induce eddy currents in bulk metals depending on the geometry of the metal and the orientation relative to the field. This problem has been characterised for various medical implants[5], for wires,[6] and for planar strips[7]. For general orientations of a single metal strip, the linearly polarized radiofrequency (RF) field stimulates eddy currents that cause signal loss at the faces and edges. These are minimised only when the plane of the strip is aligned with the RF (Figure SI1).

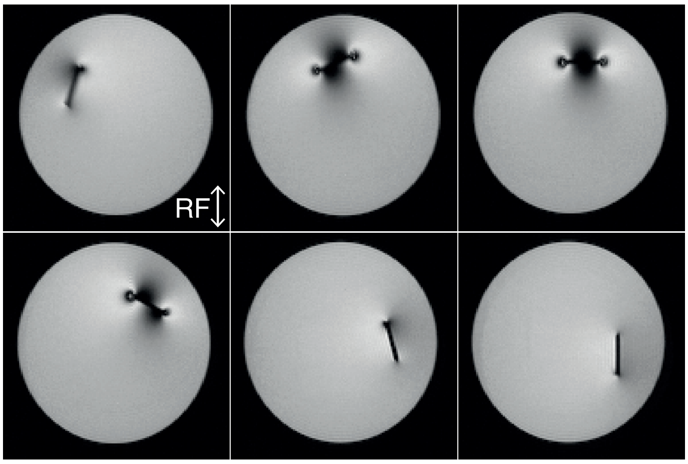

Figure SI1. **Horizontal MRI images of a cylindrical test tube containing a single metal strip.** The orientation of the linearly polarised radiofrequency (RF) field is indicated in the first image.

Electrochemistry requiring both a working electrode and a counter electrode necessitates a pair of parallel strips, and the presence of two metal structures introduces new artefacts. Figure SI2a shows horizontal and vertical images of a single metal strip in pure water, which is aligned with the RF direction, and therefore the images are distortion-free with uniform signal intensity. With the addition of a second strip (Fig. SI2b), there is signal intensity difference inside/outside the strips, and there are distortions at the strip edges that introduce additional features at the edge of the metal strips. Neither of these effects is minimised by RF power optimisation or shimming of the B_0 field. The severity of the effect varies with fluid type. When hexane is used as the medium (Fig. SI2c), the images are not affected by the same artefacts. The RF wavelength in the medium differs from the vacuum wavelength (λ_0) depending on the dielectric constant, $\lambda = \lambda_0/\sqrt{\epsilon}$. Since the wavelength for water (ε = 81, λ = 11 cm) is considerably closer to the sample length than for hexane (ε = 1.88, λ = 73 cm), it was hypothesized that the artefact could be a near-field effect that depends on the ratio of sample height to RF wavelength and decreasing the sample height from 5 cm to 2 cm effectively minimises the artefact (Figure SI2d).

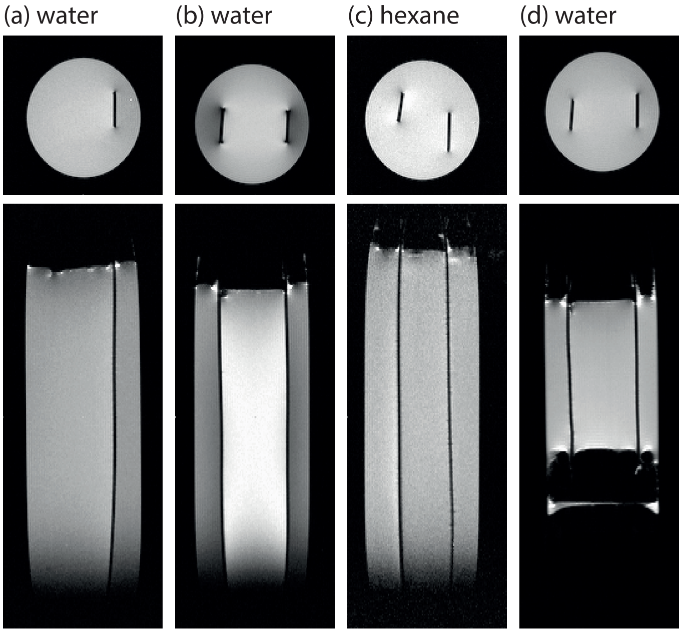

Figure SI2. **Horizontal (top) and vertical MRI images (bottom) of metal strip electrodes in a cylindrical test tube.** (a) Single electrode in deionised water. (b) Two electrodes in deionised water. (c) Two electrodes in hexane. (d) Two electrodes in deionised water, 2 cm fluid column height.

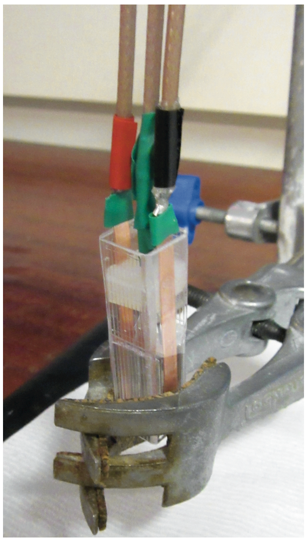

Figure SI3. **Photograph of assembled electrochemical cell.**

A photograph of the sample used is shown in Fig. SI3. Two copper strip electrodes are affixed to the walls of a semi-micro cuvette, and the electrolyte is introduced into the well between them. The height of the electrolyte fluid is 18 mm, and the artefact demonstrated in Fig. SI2 is effectively minimised. Connected to the electrodes are two shielded, non-magnetic, low susceptibility cables that are labelled with red/black tape to denote the working electrode (WE)/counter electrode (CE) respectively. A third cable is connected to the Ag/AgCl reference electrode, which is kept stably positioned with the tip immersed in the fluid using a silicone rubber plug that fits into the opening of the cell.

4. Cu^{2+} Concentration calibration

The relaxation rate ($1/T_1$) of the 0.5 M Na_2SO_4 electrolyte depends linearly on Cu^{2+} concentration, and a calibration was determined by linear least-squares fit, giving the following equation:

$$1/T_1 = (0.713\ s^{-1}\ mM^{-1})\ [Cu^{2+}] + 0.382\ s^{-1}.$$

Here, the relaxivity is r_1 = 0.713 s^{-1} mM^{-1} and the relaxation rate of the 0.5 M Na_2SO_4 electrolyte (i.e. in the absence of Cu^{2+}) is $1/T_1$ = 0.382 s^{-1} (*i.e.* T_1 = 2.62 s).

5. Optical images showing change in pH using Universal Indicator

The concentration maps in Figure 3b, and concentration profiles in Figure 4a, show a reduction in the amount of Cu^{2+}. Also, Figure 4b shows local Cu^{2+} concentrations that appear to become negative. Both observations can be explained by cathodic

reduction of oxygen with water to form hydroxide, which produces an increase in pH and a decrease in the concentration of dissolved oxygen gas. It is known[8] that pH alone does not affect the T_1 of water in the range pH 3 to 7; however, the stable species at pH > 10 are Cu_2O and $Cu(OH)_2$, both solid and therefore MRI-invisible. The change in pH was visualised by acquiring photographs of a sample cell with 6 vol-% Universal Indicator added to the electrolyte. Figure SI4 shows optical photographs of the backlit cell, confirming a transition from pH 5.5 (yellow-orange) to pH > 10 (purple), occurring at the cathode. A clear front between the low and high pH regions develops and resembles the front observed in the Cu^{2+} concentration maps (Figure 3b).

The change in pH accounts for the observation of lower Cu^{2+} concentrations than expected, and the apparently negative concentrations are due to removal of paramagnetic O_2, which increases the local T_1 and appears as a lower Cu^{2+} concentration. The amount of dissolved oxygen at atmospheric conditions is approximately 0.28 mM. With T_1 = 2.62 s for the (oxygen-containing) electrolyte and estimating[9] the oxygen relaxivity to be 153 $s^{-1}M^{-1}$ complete oxygen reduction could theoretically increase the relaxation time to a maximum of T_1 = 2.95 s. The maximum T_1 observed in the relaxation maps was indeed (2.95 ± 0.04) s, corresponding to an apparent Cu^{2+} concentration of –0.061 mM. This occurred near the bottom of the cathode, with variably less effect higher up in the cell, such as in the profiles of Figure 3b.

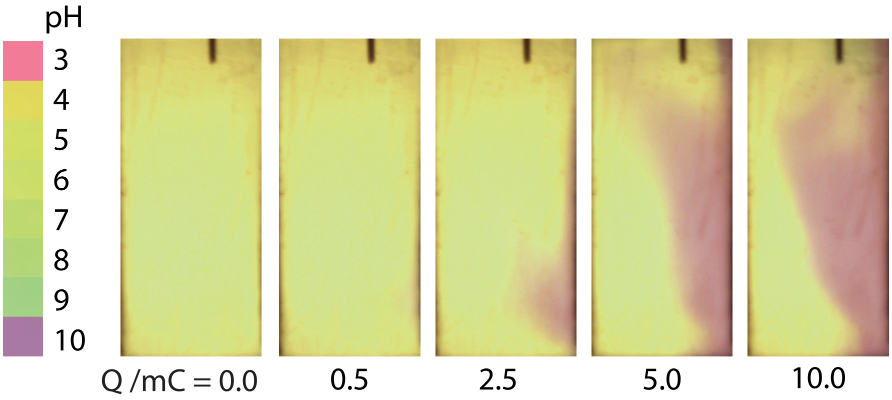

Figure SI4. **Photographs of a cell containing 0.5 M Na_2SO_4 with 6 %-vol universal indicator.** The cell construction is the same as in Fig. 3, and the net charge (Q) delivered by the potentiostat is given below.

6. Electrochemistry

The alignment of the copper strips was chosen to minimise both RF absorption and distortions of the primary B_0 field, but this orientation also minimises magnetic flux changes from the field gradients used for imaging, which may induce transient voltages in the circuit when switching on/off. The magnetic field gradients of the MRI experiment produced no discernable fluctuations in the OCP.

7. Concentration calibration for 3D mapping

The images are normalised, so that variations in signal intensity in each image are only through variations in Cu^{2+} concentration. The relationship between the normalised signal intensity (S), for the 3D, spin-echo MRI images shown in figure 5, is given by equation (S1) and is dependent on the local T_1 relaxation time, which is dependent on the concentration of Cu^{2+}, the repetition time (TR) and equilibrium intensity (S_0).

$$S = S_0\,(1 - \exp(-TR/T_1)) \qquad \text{(S1)}$$

S_0 is the signal intensity for the solution where $TR > 5T_1$ and by plotting S against $(1 - \exp(-TR/T_1))$, for a range of solutions with varying Cu^{2+} concentrations, a straight line is produced, allowing simple mapping between S and T_1, which leads to the determination of a $[Cu^{2+}]$. Figure SI5 shows (a) the images used for the calibration, and (b) the calibration data, which was fitted to equation $S1$ using linear least-squares. Maps of Cu^{2+} were then obtained by converting signal intensity into Cu^{2+} concentration.

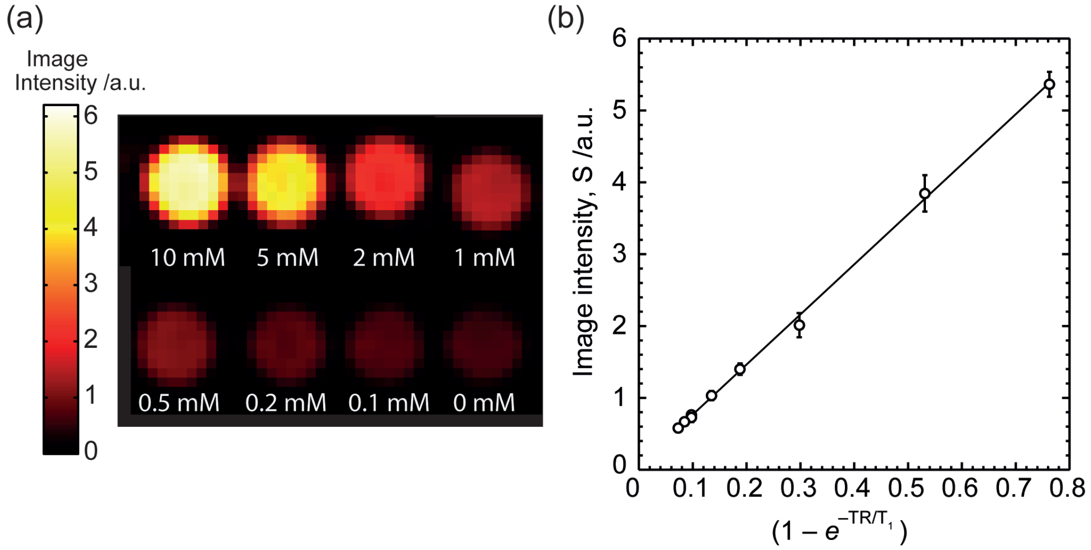

Figure SI5. **Calibration data for 3D concentration mapping.** (a) Tubes containing solutions of 0.5 M Na_2SO_4 with varying concentrations of $CuSO_4$. The concentration of $CuSO_4$ in each tube is indicated in the image and has the following T_1 relaxation times: 0.145 s (10 mM), 0.26 s (5 mM), 0.57 s (2 mM), 0.96 s (1 mM), 1.38 s (0.5 mM), 1.94 s (0.2 mM), 2.25 s (0.1 mM), 2.62 s (0 mM). (b) Calibration plot of image intensity against T_1 relaxation time.

1 Britton, M. M. Magnetic resonance imaging of chemistry. *Chem. Soc. Rev.* **39**, 4036-4043, (2010).

2 Hertz, H. G. Microdynamic behaviour of liquids as studied by NMR relaxation times. *Prog. Nucl. Magn. Reson. Spec.* **3**, 159-230, (1967).

3 Lauffer, R. B. Paramagnetic Metal Complexes as Water Proton Relaxation Agents for NMR Imaging: Theory and Design. *Chemical Reviews* **87**, 901-907, (1987).

4 Hennig, J., Naureth, A. & Friedburg, H. RARE imaging: A fast imaging method for clinical MR. *Magn. Reson. Med.* **3**, 823-833, (1986).

5 Graf, H., Steidle, G., Martirosian, P., Lauer, U. A. & Schick, F. Effects on MRI due to altered rf polarization near conductive implants or instruments. *Medical Physics* **33**, 124-127, (2006).

6 Bennett, L. H., Wang, P. S. & Donahue, M. J. Artifacts in magnetic resonance imaging from metals. *Journal of Applied Physics* **79**, 4712-4714, (1996).

7 Britton, M. M., Bayley, P. M., Howlett, P. C., Davenport, A. J. & Forsyth, M. In Situ, Real-Time Visualization of Electrochemistry Using Magnetic Resonance Imaging. *J. Phys. Chem. Lett.* **4**, 3019-3023, (2013).

8 Balcom, B. J., Carpenter, T. A. & Hall, L. D. Spatial and Temporal Visualization of a Ph-Dependent Complexation Equilibrium by Nuclear-Magnetic-Resonance Imaging. *Can J Chem* **70**, 2693-2697, (1992).

9 Wang, Z. J. *et al.* Urinary Oxygen Tension Measurement in Humans Using Magnetic Resonance Imaging. *Acad Radiol* **15**, 1467-1473, (2008).
